# Comparison of Arrhythmia Prevalence and Incidence in Adult Patients with Lateral Tunnel and Extra-Cardiac Fontan Circulation

**DOI:** 10.1007/s00246-025-03950-1

**Published:** 2025-08-20

**Authors:** Andrew M. Freddo, Antara Mondal, Alexis Z. Tomlinson, Molly Eron, Srinivas Denduluri, Isabella Farkas, Sara Partington, Emily Ruckdeschel, Allison L. Tsao, Constantine D. Mavroudis, Muhammad Nuri, Stephanie Fuller, Yuli Y. Kim, Sumeet Vaikunth

**Affiliations:** 1 Division of Cardiology, Department of Pediatrics, Children’s Hospital of Philadelphia, 3401 Civic Center Boulevard, Philadelphia, PA 19104 USA; 2 Division of Cardiovascular Medicine, Department of Medicine, Hospital of the University of Pennsylvania, Philadelphia, PA USA

**Keywords:** Congenital heart disease, Arrhythmia, Fontan, Single ventricle, Adult congenital heart disease

## Abstract

Although patients with total cavopulmonary connection (TCPC) Fontan are known to have a lower incidence of arrhythmias compared with atriopulmonary Fontan, there is debate about long-term prevalence of arrhythmia in the TCPC population or about the differences in arrhythmia burden between patients with lateral tunnel (LT) and extra-cardiac (EC) Fontan circulations. A retrospective cohort of 341 patients with LT and EC Fontan operations was identified in a single adult congenital heart disease (ACHD) center. Arrhythmias and related procedures were recorded. Logistic regression was used to determine factors affecting arrhythmia prevalence. Arrhythmia incidence was compared using interval-censored Cox regression. Of 341 patients, 254 (74.5%) had LT Fontan and 87 (25.5%) had EC Fontan. Patients with LT Fontan were older at their last ACHD follow-up (29 versus 25 years). Arrhythmia occurred in 52.2% of patients, 57.9% of the LT and 35.6% of the EC Fontan group. Compared to EC Fontan patients, patients with LT were both more likely to have an arrhythmia (OR 1.84, 95% CI 1.04–3.24) and at a higher risk of developing a new arrhythmia (HR 1.67, 95% CI 1.10–2.54). Patients with LT had more cardioversions, ablations, and revisions compared with EC Fontan patients. However, a similar proportion in each group were treated with antiarrhythmics and had a pacemaker. We summarize arrhythmia burden in a large single-center adult cohort of patients with TCPC Fontan, demonstrating increased arrhythmia in patients with LT. This work can inform screening protocols and risk assessment for this growing population.

**Supplementary Information:**

The online version contains supplementary material available at 10.1007/s00246-025-03950-1.

## Introduction

The total cavopulmonary connection (TCPC) was developed in 1988 by de Leval in an effort to improve efficiency of the Fontan pathway and reduce arrhythmia burden [1]. With its implementation, the proportion of arrhythmia decreased from 45–65% to 14–25% in these patients, though these largely represent historical data from older patient cohorts [2]. TCPC was initially performed using a lateral tunnel (LT), creating a pathway through the atrium to allow for inferior vena cava flow to reach the pulmonary arteries. However, concerns existed that significant suture line burden within the atrium increased the risk of both short- and long-term arrhythmia. The extra-cardiac (EC) Fontan was developed soon thereafter as an attempt to minimize atrial manipulation [3]. Although some studies show similar rates of arrhythmias between LT and EC Fontan operations [4], many studies demonstrate reduced arrhythmia burden through late childhood in patients with EC Fontan compared with patients with LT Fontan [5, 6]. However, these studies are limited in scope because of the relatively short follow-up time. Recent reports of improved hemodynamics in the LT Fontan compared with EC Fontan [7, 8] are leading some to reconsider performing LT Fontan instead of EC Fontan. Here we report prevalence and incidence of arrhythmias in adults with either a LT or an EC Fontan followed at our institution and determine the number of EP procedures these patients have undergone.

## Methods

### Data Source and Study Design

A retrospective single-center cohort was identified by searching the Electronic Medical Record (EMR) for adults greater than or equal to 18 years of age using ICD-9 and ICD-10 codes indicating Fontan circulation (Supplemental Table 1) who had at least one encounter in the outpatient ACHD clinic between January 1, 2009 and December 31, 2023. Additional clinical data abstracted from the EMR included: demographics, cardiac diagnosis, details of Fontan operation, cardiac and extra-cardiac comorbidities, and electrophysiologic procedures. For cardiac and extra-cardiac comorbidities, these were noted if listed in the patient’s problem list or provider documentation. Fontan revision was defined based on provider documentation, and included surgical revision, catheter-based interventions, hepatic vein inclusion, and conversion of LT to EC Fontan. Arrhythmias were classified as bradyarrhythmias (including sinus node dysfunction, high-degree atrioventricular block, and junctional rhythm) and tachyarrhythmias (including atrial and ventricular arrhythmias) and were reported based on clinical documentation and Holter monitor, when available. Isolated asymptomatic premature atrial or ventricular contractions were excluded. Patients were reported as being prescribed an antiarrhythmic medication if they had any of the following medications prescribed as an outpatient: amiodarone, atenolol, diltiazem, dofetilide, flecainide, mexiletine, nadolol, propafenone, propranolol, sotalol, or verapamil. Follow-up was defined as the time from first clinic visit until either last confirmed contact in the EMR (up to and including January 31, 2024) or date of heart transplant or death. This study was approved by the University of Pennsylvania IRB 827550.

### Primary Endpoint

The primary endpoint was prevalence of arrhythmia.

### Secondary Endpoint

The secondary endpoint was development of a new arrhythmia.

### Data Analysis

Descriptive statistics were reported as frequencies and percentages for categorical variables, and median and interquartile range (IQR) for continuous variables. Categorical variables were compared with Chi Square or Fisher’s Exact Test.

#### Aim 1

Patient factors associated with presence of arrhythmia were determined using logistic regression. Factors found to be associated with presence of arrhythmia on univariable analysis with p-value less than 0.10 were incorporated into a multivariable logistic regression model.

#### Aim 2

An interval-censored Cox regression model was performed to determine association between patient factors and time to development of new arrhythmia. For these patients, time to new arrhythmia was calculated from the date of Fontan surgery to the date of new arrhythmia. If patients had arrhythmia noted at their first ACHD visit, they were modeled as left-censored. It was assumed that the arrhythmia developed between Fontan surgery and establishment of ACHD care. Patients who achieved their first reported arrhythmia during ACHD follow-up were uncensored. Patients who had no documented arrhythmia were modeled as right-censored. For these patients, observation time was calculated from the date of Fontan surgery to the date of last ACHD contact (Supplemental Fig. 1). Like earlier arrhythmia incidence work [9], patients who did not develop arrhythmia, but experienced death or heart transplant were censored accordingly. Upon detailed examination, no patient deaths were directly attributable to new arrhythmias. Of the 24 patients who died or underwent heart transplant, only 3 had an unknown cause of death and one had recurrence of an existing tachyarrhythmia prior to death.

Statistical analysis was performed using STATA version 18 (StataCorp, College Station, TX). The proportional hazards assumption was assessed for all Cox models. Significance for all statistical tests was defined as *p* < 0.05.

## Results

### Cohort Characteristics

Table 1 provides a summary of the 341 patients included in the analysis. In total, 254 patients had LT Fontan and 87 patients had EC Fontan. Patients with LT Fontan were older (median age 24 years at first ACHD visit) compared with patient with EC Fontan (median age 22) (*p* < 0.001) and had Fontan completion during an earlier surgical era, with 68.9% of operations occurring between 1991–2000, compared with EC Fontan patients, with 51.7% of operations occurring in 2001 or later (*p* < 0.001). Although underlying primary cardiac diagnosis differed between the groups (*p* = 0.005), predominant single ventricle morphology did not (*p* = 0.918). The proportion of patients with a fenestration and who underwent Fontan revision also did not differ significantly between the groups. Nearly all revisions (18/19) were surgical, and 8 patients with LT Fontan were converted to EC Fontan. Median follow-up time was 3.63 years, longer for LT Fontan patients (4.25 years) and shorter for EC Fontan patients (2.82 years) (*p* < 0.001).

**Table 1:** Cohort characteristics stratified by type of Fontan operation

Category	*N* (%) *or* Median [IQR]			*p*-value
	Overall (*N* = 341)	LT (*N* = 254)	EC (*N* = 87)	
Male	189 (55.4)	151 (59.5)	28 (43.7)	0.011
Age at first ACHD visit, years	23 [21–27]	24 [21–28]	22 [20–24]	< 0.001
Age at last follow-up, years	28 [25–32]	29 [26–33]	25 [23–28]	< 0.001
Follow-up time, years	3.63 [1.60–6.50]	4.25 [1.99–7.04]	2.82 [1.00–4.18]	< 0.001
Race				0.051
Asian	10 (2.9)	5 (2.0)	5 (5.8)	
Black	41 (12.0)	30 (11.8)	11 (12.6)	
White	235 (68.9)	183 (72.1)	52 (59.8)	
Other	14 (4.1)	7 (2.8)	7 (8.1)	
Unknown	41 (12.0)	29 (11.4)	12 (13.8)	
Hispanic/Latino ethnicity	16 (4.7)	7 (2.8)	9 (10.3)	0.004
Primary cardiac diagnosis				0.005
Tricuspid atresia	65 (19.1)	45 (17.7)	20 (23.0)	
Hypoplastic left heart syndrome	107 (31.4)	90 (35.4)	17 (19.5)	
Double-outlet right ventricle	49 (14.4)	37 (14.6)	12 (13.8)	
Double-inlet left ventricle	48 (14.1)	40 (15.8)	8 (9.2)	
Unbalanced AV canal	37 (10.9)	21 (8.3)	16 (18.4)	
Pulmonary atresia/intact ventricular septum	19 (5.6)	12 (4.7)	7 (8.1)	
Other	16 (4.7)	9 (3.5)	7 (8.1)	
Ventricular morphology				0.918
Single left ventricle	140 (41.1)	103 (40.6)	37 (42.5)	
Single right ventricle	175 (51.3)	132 (52.0)	43 (49.4)	
Mixed ventricular morphology	26 (7.6)	19 (7.5)	7 (8.1)	
Heterotaxy	45 (13.2)	24 (9.5)	21 (24.1)	< 0.001
Fenestrated	235 (68.9)	175 (68.9)	60 (69.0)	0.991
Age at Fontan, months	25.99 [20.34–41.10]	25.00 [19.55–39.03]	32.33 [22.50–47.14]	< 0.001
Fontan revision	19 (5.6)	15 (5.9)	4 (4.6)	0.791
Fontan completion date				
1976–1990	45 (13.2)	43 (16.9)	2 (2.3)	< 0.001
1991–2000	215 (63.1)	175 (68.9)	40 (46.0)	
2001–2014	81 (23.8)	36 (14.2)	45 (51.7)	

### Observed Arrhythmias

Overall, 178 patients (52.2%) had at least one type of arrhythmia before or during the study period (Table 2). Tachyarrhythmia occurred in 136 (39.9%) and bradyarrhythmia in 86 (25.2%). The most common tachyarrhythmia was atrial flutter/intra-atrial reentrant tachycardia (IART), which occurred in 43 (12.6%) patients overall. The most common bradyarrhythmia was sinus node dysfunction, which occurred in 66 (19.4%) patients.

**Table 2:** Summary of reported arrhythmias with the patient population

	Entire cohort (*N* = 341)	LT Fontan (*N* = 254)	EC Fontan (*N* = 87)
Any arrhythmia	178 (52.2)	147 (57.9)	31 (35.6)
Bradyarrhythmia	86 (25.2)	71 (28.0)	15 (17.2)
Tachyarrhythmia	136 (39.9)	114 (44.9)	22 (25.3)
Atrial tachyarrhythmia	121 (35.4)	102 (40.2)	19 (21.8)
Atrial flutter/IART	43 (12.6)	41 (16.1)	2 (2.3)
Atrial fibrillation	15 (4.4)	14 (5.5)	1 (1.2)
Other AT	20 (5.9)	17 (6.7)	3 (3.5)
SVT	57 (16.7)	46 (18.1)	11 (12.6)
Ventricular tachyarrhythmia	37 (10.9)	30 (11.8)	7 (8.0)
Other arrhythmia	10 (2.9)	6 (2.4)	4 (4.6)

Percentages calculated based on type of Fontan operation. N.B. Patients may be in multiple categories if more than one arrhythmia is reported

*AT* atrial tachycardia, *IART* intra-atrial reentrant tachycardia, *SVT* supraventricular tachycardia

### Factors Associated with Arrhythmias

Factors associated with presence of arrhythmia were determined and are summarized in Table 3. On univariable analysis, age at last ACHD contact (OR 1.137, 95% CI 1.085–1.190), presence of a LT Fontan (2.48, 1.50–4.11), and age at Fontan (1.008, 1.002–1.014) were associated with arrhythmia. In a multivariable analysis, age at last ACHD contact (1.112, 1.055–1.172) and presence of LT Fontan (1.84, 1.04–3.24) remained associated with the presence of arrhythmia.

**Table 3:** Factors affecting presence of any arrhythmia

	Univariable			Multivariable		
Characteristic	Odds ratio	95% CI	*p*-value	Odds ratio	95% CI	*p*-value
Male	1.23	0.80–1.89	0.344			
Age at last ACHD contact	1.137	1.085–1.190	< 0.001	1.112	1.055–1.172	< 0.001
Ventricular morphology (ref = single LV)						
Single RV	0.97	0.62–1.51	0.880			
Mixed ventricular morphology	0.89	0.39–2.06	0.789			
Heterotaxy	1.79	0.93–3.43	0.080			
Lateral tunnel Fontan	2.48	1.50–4.11	< 0.001	1.84	1.04–3.24	0.036
History of revision	2.70	0.95–7.66	0.063			
Age at Fontan, months	1.008	1.002–1.014	0.009	1.003	0.996–1.011	0.390

Similar analyses were performed for bradyarrhythmias (Supplemental Table 2) and tachyarrhythmias (Supplemental Table 3). For bradyarrhythmias, heterotaxy (0.25, 0.09–0.73), presence of a LT Fontan (1.86, 1.00–3.46), and history of Fontan revision (4.53, 1.75–11.67) were found to be significant on univariable analysis; heterotaxy and history of Fontan revision remained significant on multivariable analysis. Increased age at last ACHD contact (1.181, 1.123–1.241), heterotaxy (2.57, 1.35–4.89), presence of a LT Fontan (2.41, 1.40–4.14), and age at Fontan (1.012, 1.006–1.019) were associated with tachyarrhythmia on univariable analysis. On multivariable analysis for tachyarrhythmia, LT Fontan was nearly significant (1.90, 0.99–3.67) and both age at last contact and heterotaxy remained significant.

### Development of New Arrhythmia

During follow-up with the ACHD program, 39 patients (11.4%) developed a new arrhythmia not previously reported at initial ACHD contact. This occurred more frequently in patients with LT Fontan (34, 13.4% of LT Fontan patients) than with EC Fontan (5, 5.7% of EC Fontan patients), however, did not reach significance (*p* = 0.053). New atrial tachyarrhythmias were much more common (Table 4).

**Table 4:** Development of new arrhythmia during ACHD follow-up

	Entire cohort (*N* = 39)	LT Fontan (*N* = 34)	EC Fontan (*N* = 5)
Bradyarrhythmia	3 (7.7)	3 (8.8)	0 (0.0)
Tachyarrhythmia	30 (76.9)	27 (79.4)	3 (60.0)
Atrial tachyarrhythmia	31 (79.5)	27 (79.4)	4 (80.0)
Atrial flutter/IART or atrial fibrillation	13 (33.3)	12 (35.3)	1 (20.0)
SVT	14 (35.9)	12 (35.3)	2 (40.0)
Other AT	4 (10.3)	3 (8.8)	1 (20.0)
Ventricular tachyarrhythmia	5 (12.8)	4 (11.8)	1 (20.0)

Percentages are out of patients developing a new arrhythmia in each column. N.B. 2 EC and 4 LT patients had a reported tachyarrhythmia prior to establishing ACHD care and developed an additional tachyarrhythmia during follow-up, so the sum of patients with new atrial and new ventricular tachyarrhythmias exceeds the number of patients with new tachyarrhythmia. One patient with LT Fontan had development of an unspecified tachyarrhythmia, and another patient with LT Fontan had development of both an atrial and ventricular tachyarrhythmia reported at the same time

These data were incorporated into an interval-censored Cox regression model (Table 5), which demonstrated an association between increased hazard of development of arrhythmia and presence of an LT Fontan (HR 1.58, 95% CI 1.06–2.37), heterotaxy (1.56, 1.10–2.23), history of Fontan revision (1.69, 1.15–2.49), and older age at Fontan (1.005, 1.003–1.008) on univariable analysis, of which only heterotaxy did not achieve significance on multivariable analysis.

**Table 5:** Survival analysis for development of late-onset arrhythmia

	Univariable			Multivariable		
Characteristic	Hazard ratio	95% CI	*p*-value	Hazard ratio	95% CI	*p*-value
Male	1.13	0.84–1.54	0.418			
Ventricular morphology (ref = single LV)						
Single RV	0.92	0.67–1.26	0.609			
Mixed ventricular morphology	0.84	0.48–1.48	0.551			
Heterotaxy	1.57	1.10–2.23	0.013	1.37	0.92–2.04	0.120
Lateral tunnel Fontan	1.58	1.06–2.36	0.025	1.67	1.10–2.54	0.015
History of revision	1.69	1.15–2.49	0.007	1.77	1.20–2.62	0.004
Age at Fontan, months	1.005	1.003–1.008	< 0.001	1.005	1.002–1.007	< 0.001

### Arrhythmia Treatment

The number of patients undergoing EP procedures was ascertained (Table 6). In total, 32 patients (18.0%) had at least one cardioversion. Of the LT Fontan patients with arrhythmia, 30 (20.4%) had a cardioversion; of the EC Fontan patients, 2 (6.5%) had a cardioversion. Ablation was performed in 23 patients (12.9%). This occurred in 21 (14.3%) of the LT Fontan patients with arrhythmia and 2 (6.5%) of the EC Fontan patients with arrhythmia. MAZE procedure was performed in 4 patients (2.2%), all with LT Fontan, 3 of which were concurrent with Fontan revision.

**Table 6:** Treatments and interventions for arrhythmias in patients included in the cohort, as reported in their history

	Total (*N* = 178)	LT Fontan (*N* = 147)	EC Fontan (*N* = 31)
DCCV	32 (18.0)	30 (20.4)	2 (6.5)
Ablation	23 (12.9)	21 (14.3)	2 (6.5)
MAZE	4 (2.2)	4 (2.7)	0 (0.0)
Fontan revision	14 (7.9)	12 (8.2)	2 (6.5)
Revision for arrhythmia	5 (2.8)	5 (3.4)	0 (0.0)
Pacemaker/ICD	62 (34.8)	52 (35.4)	10 (32.3)
Antiarrhythmic medication	63 (35.4)	53 (36.1)	10 (32.3)

Percentages are based on the patients in each category with arrhythmia. Differences between LT and EC Fontan groups were all insignificant by Fisher’s Exact Test

*DCCV* DC cardioversion, *ICD* implantable cardioverter-defibrillator

A pacemaker or implantable cardioverter-defibrillator (ICD) was implanted in 62 (34.8%) patients; 52 (35.4%) LT Fontan patients and 10 (32.3%) EC Fontan patients. Nearly all patients had epicardial systems: only 6 patients (all LT Fontan) had a transvenous system, with an additional 3 LT Fontan patients having a history of a transvenous system that was later converted to an epicardial one. Of the cohort, 63 (35.4%) patients were prescribed antiarrhythmic medication; 53 (36.1%) of LT Fontan patients and 10 (32.3%) of EC Fontan patients.

## Discussion

Here we report one of the largest long-term studies of late arrhythmia burden in patients undergoing LT and EC Fontan operations. We confirm a higher burden of arrhythmia in the LT versus EC Fontan patients and reveal that the burden of arrhythmia in EC Fontan is greater than previously described. We also find that this difference is more pronounced for tachyarrhythmia than for bradyarrhythmia.

### Presence of Arrhythmia

Whereas previous studies are limited to the evaluation of children and adolescents [4–6], our study is the first direct comparison of LT and EC Fontan patients in adulthood. A recent study of over 300 EC Fontan patients at a single-center [9] was one of the first studies to highlight the risk of arrhythmia in the aging EC Fontan population. With a slightly shorter median follow-up time post-Fontan (15 years) compared to our study, just over one quarter of patients having an arrhythmia during follow-up. However, there was no comparison to LT Fontan patients.

We found that although atrial tachyarrhythmias are the most common sub-type of arrhythmia in both LT and EC Fontan groups, the type of atrial tachyarrhythmia differs. In LT Fontan patients, both atrial flutter/IART and SVT are seen with similar frequencies (16.1 and 18.1%, respectively), though within the EC Fontan group, atrial flutter/IART was less common than SVT (2.3% and 12.6%, respectively). While there may be an age-related factor that explains this difference, this is likely in large part due to the atrial suture lines required in the LT Fontan. Ventricular tachyarrhythmia is reported at a similar rate in this study (8.0%) compared with Di Mambro’s study of arrhythmia in the EC Fontan patient population (9.5%) [9].

Though not related to the presence of bradyarrhythmia, age has a strong relationship with tachyarrhythmia, which has also been described in meta-analyses of younger Fontan patients [10]. This may be because cumulative changes to atrial size and histology may increase risk of tachyarrhythmia with time [11]. In contrast, patients with Fontan circulation who do not have additional surgical manipulation near the sinus and/or AV nodes should not have an increased risk of developing bradyarrhythmias with advancing age.

### Development of Arrhythmia with Time

An important consideration is not only the presence of arrhythmia, but the cumulative risk of development of arrhythmia over time. This has been well-described in the LT Fontan population [5], and here we provide the first direct comparison of incidence of late arrhythmia in EC and LT adult Fontan patients. Because of our study design, we are not able to capture exact dates of arrhythmia onset prior to patients establishing care with our ACHD clinic. However, we account for this limitation by utilizing time-to-event analysis with left censoring, which allows us to incorporate data for patients presenting to our clinic who already developed an arrhythmia. Especially because our EC and LT Fontan populations differ significantly in age, this allows us to consider development of arrhythmia through the lifespan, not only during ACHD follow-up, and more directly compare the EC and LT Fontan patient populations. With this model, we find that patients with LT Fontan have a higher HR of arrhythmia incidence.

We also show that, regardless of Fontan type, there is an increased hazard of developing arrhythmia in patients who had their Fontan operations at a later age. This has been seen in other studies evaluating late arrhythmias [12, 13], but not others [14]. This risk may be a result of increased volume loading of the single ventricle prior to cavopulmonary anastomosis, resulting in decreased ventricular compliance and elevated ventricular and atrial pressures. In turn, this could cause atrial fibrosis and increase arrhythmia risk. We also find that history of revision has a strong association with development of arrhythmia; although this is a heterogenous category spanning various surgical and percutaneous interventions, it highlights the association between manipulation of the Fontan anatomy and risk of arrhythmia. There also are likely many cases when arrhythmia preceded the revision, increasing this association.

### Treatments and Interventions for Arrhythmia

Presence or development of arrhythmia indicates potential worsening Fontan hemodynamics. For example, in sinus node dysfunction or high-degree AV block, loss of atrioventricular synchrony can impede cardiac output [15]. There is an established connection between arrhythmia and increased comorbidities, medications, procedures, and both hospitalizations and hospital lengths of stay [16].

Additionally, procedural interventions have a lower rate of success in patients with Fontan palliation compared with other types of CHD [11]. Of patients with arrhythmia, we find that 18.1% of patients had at least one DC cardioversion and 14.3% had at least one ablation. Most commonly, these were for atrial flutter/IART and/or atrial fibrillation, consistent with previous studies [2]. Unfortunately, we were underpowered to meaningfully compare rates of this in EC and LT Fontan patients with these data, though most patients undergoing procedures had an LT Fontan. Although we did not assess directly for recurrence of arrhythmia in our cohort, previous studies have found an increased risk of recurrence of atrial arrhythmias in adult Fontan patients who have a history of atrial arrhythmia [17].

Interestingly, the proportion of patients with arrhythmia who had pacemakers was nearly identical between the LT and EC Fontan groups. As most patients had a pacemaker placed for bradyarrhythmia, this follows with the lack of association between bradyarrhythmia and Fontan type. It is also important to note that patients almost exclusively had epicardial pacemaker leads instead of transvenous systems, likely because of the limitations of manipulating transvenous leads through a total cavopulmonary connection [2]. There also is a nearly identical proportion of patients in the LT and EC Fontan groups treated with antiarrhythmic medication. It is unclear why prescription of antiarrhythmics is so similar between these patients, though it may be due to attempting antiarrhythmic treatment as a first line, not having a severe enough arrhythmia burden to warrant more invasive measures, or inability to map and ablate an arrhythmia because of the Fontan anatomy.

### Limitations

We are limited as this study is retrospective, hence we rely on chart review and provider documentation for data regarding arrhythmia. Additionally, we do not have surgical data from all patients, including surgeon and conduit size (for EC Fontan) to make inferences based on these factors. Because our study is based at an ACHD center, where many patients are referred from pediatric cardiology programs or other hospital systems, we are unable to pinpoint exact dates of arrhythmia onset before establishing ACHD care which may result in exclusion of some earlier arrhythmias. Left-censoring in our time-to-event analysis was utilized to account for these limitations.

## Conclusion

We describe one of the first studies comparing arrhythmia burden in adult patients with long-standing LT and EC Fontan circulation. LT Fontan appears to carry a higher risk of presence or development of any arrhythmia. However, patients living with EC Fontan have a higher than previously described arrhythmia burden. Future studies can incorporate additional longitudinal data to determine potential screening and treatment recommendations for these patients.

## Supplementary Information

Below is the link to the electronic supplementary material.

Supplementary file1 (DOCX 56 KB)

Supplementary file2 (DOCX 17 KB)

Supplementary file3 (DOCX 18 KB)

Supplementary file4 (DOCX 18 KB)

## Data Availability

No datasets were generated or analysed during the current study.
