# Supplementary material for: Comparison of Arrhythmia Prevalence and Incidence in Adult Patients with Lateral Tunnel and Extra-Cardiac Fontan Circulation
Source: Pediatr Cardiol. 2025 Aug 20;47(4):1658–65. doi: 10.1007/s00246-025-03950-1 (PMC12945893; doi:10.1007/s00246-025-03950-1)

Comparison of arrhythmia prevalence and incidence in adult patients with lateral tunnel and extra-cardiac Fontan circulation

Journal: Pediatric Cardiology

Andrew M Freddo, MD, PhD^a^ ([andrew.freddo@osumc.edu](mailto:andrew.freddo@osumc.edu))

Antara Mondal, MS^a^

Alexis Z Tomlinson, PhD^a^

Molly Eron, BS^b^

Srinivas Denduluri, PhD^b^

Isabella Farkas, BA^b^

Sara Partington, MD^a,b^

Emily Ruckdeschel, MD^a,b^

Allison L Tsao, MD^a,b^

Constantine D Mavroudis, MD, MSc, MTR^a^

Muhammad Nuri, MD^a^

Stephanie Fuller, MD, MS^a^

Yuli Y Kim, MD^a,b^

Sumeet Vaikunth, MD, MEd^a,b^

1. Division of Cardiology, Department of Pediatrics, Children’s Hospital of Philadelphia, Philadelphia, Pennsylvania, USA
2. Division of Cardiovascular Medicine, Department of Medicine, Hospital of the University of Pennsylvania, Philadelphia, Pennsylvania, USA

**Supplemental Figure 1: Schema for defining new arrhythmia endpoint.** Timeline summarizing new arrhythmia endpoint as defined in time-to-event analysis. Solid line indicates time patients were followed in the study, with dashed line indicating time before establishing care in the ACHD clinic. Patients with arrhythmia present at their first visit were left-censored. Patients who reached the end of follow-up (either last ACHD contact, transplant, or death) without an arrhythmia were right-censored. Patients with reported arrhythmia during ACHD follow-up were not censored.


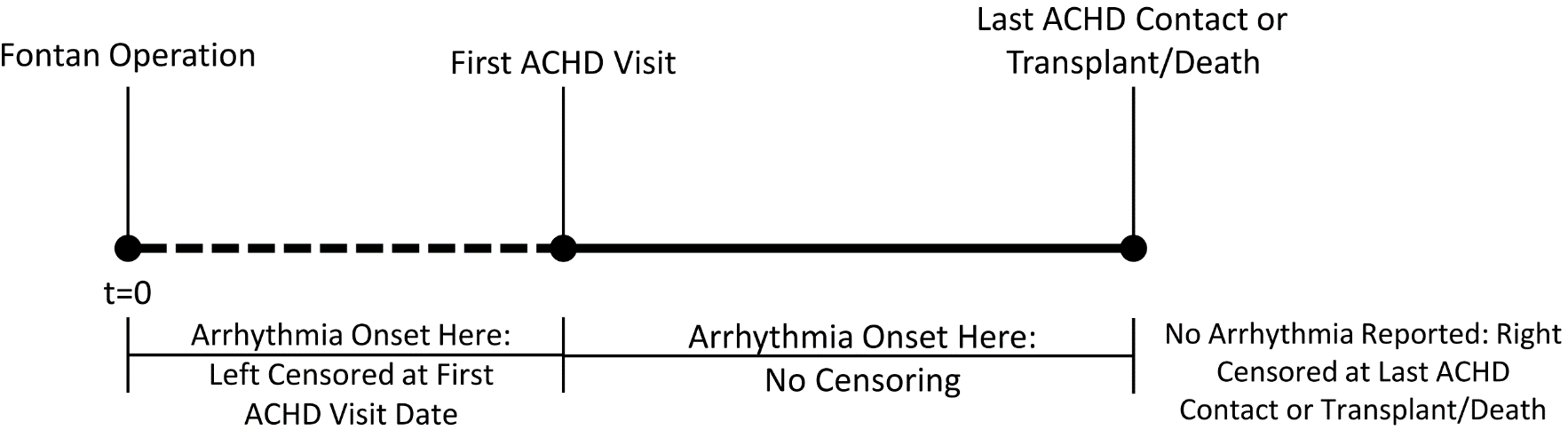

Supplement: Supplementary file 1 — Supplementary file1 (DOCX 56 KB) [file 246_2025_3950_MOESM1_ESM.docx]
