# Supplementary material for: Comparison of Arrhythmia Prevalence and Incidence in Adult Patients with Lateral Tunnel and Extra-Cardiac Fontan Circulation
Source: Pediatr Cardiol. 2025 Aug 20;47(4):1658–65. doi: 10.1007/s00246-025-03950-1 (PMC12945893; doi:10.1007/s00246-025-03950-1)
Supplement: Supplementary file 2 — Supplementary file2 (DOCX 17 KB) [file 246_2025_3950_MOESM2_ESM.docx]

Comparison of arrhythmia prevalence and incidence in adult patients with lateral tunnel and extra-cardiac Fontan circulation

Journal: Pediatric Cardiology

Andrew M Freddo, MD, PhD^a^ ([andrew.freddo@osumc.edu](mailto:andrew.freddo@osumc.edu))

Antara Mondal, MS^a^

Alexis Z Tomlinson, PhD^a^

Molly Eron, BS^b^

Srinivas Denduluri, PhD^b^

Isabella Farkas, BA^b^

Sara Partington, MD^a,b^

Emily Ruckdeschel, MD^a,b^

Allison L Tsao, MD^a,b^

Constantine D Mavroudis, MD, MSc, MTR^a^

Muhammad Nuri, MD^a^

Stephanie Fuller, MD, MS^a^

Yuli Y Kim, MD^a,b^

Sumeet Vaikunth, MD, MEd^a,b^

1. Division of Cardiology, Department of Pediatrics, Children’s Hospital of Philadelphia, Philadelphia, Pennsylvania, USA
2. Division of Cardiovascular Medicine, Department of Medicine, Hospital of the University of Pennsylvania, Philadelphia, Pennsylvania, USA

Supplemental Table 1: ICD9/10 codes utilized for patient identification.

| **Diagnosis Name** | **ICD9 Code** | **ICD10 Code** |
| --- | --- | --- |
| Status post Fontan procedure | V45.89 | Z98.890 |
| S/P Fontan procedure | V45.89 | Z98.890 |
| Post-Fontan protein-losing enteropathy | 579.8 | K90.49, Z98.890 |
| Status post Fontan operation | V45.89 | Z98.890 |
| Status post hemi-Fontan operation | V45.89 | Z98.890 |
| S/P hemi-Fontan operation | V45.89 | Z98.890 |
