# Supplementary material for: Comparison of Arrhythmia Prevalence and Incidence in Adult Patients with Lateral Tunnel and Extra-Cardiac Fontan Circulation
Source: Pediatr Cardiol. 2025 Aug 20;47(4):1658–65. doi: 10.1007/s00246-025-03950-1 (PMC12945893; doi:10.1007/s00246-025-03950-1)
Supplement: Supplementary file 4 — Supplementary file4 (DOCX 18 KB) [file 246_2025_3950_MOESM4_ESM.docx]

Comparison of arrhythmia prevalence and incidence in adult patients with lateral tunnel and extra-cardiac Fontan circulation

Journal: Pediatric Cardiology

Andrew M Freddo, MD, PhD^a^ ([andrew.freddo@osumc.edu](mailto:andrew.freddo@osumc.edu))

Antara Mondal, MS^a^

Alexis Z Tomlinson, PhD^a^

Molly Eron, BS^b^

Srinivas Denduluri, PhD^b^

Isabella Farkas, BA^b^

Sara Partington, MD^a,b^

Emily Ruckdeschel, MD^a,b^

Allison L Tsao, MD^a,b^

Constantine D Mavroudis, MD, MSc, MTR^a^

Muhammad Nuri, MD^a^

Stephanie Fuller, MD, MS^a^

Yuli Y Kim, MD^a,b^

Sumeet Vaikunth, MD, MEd^a,b^

1. Division of Cardiology, Department of Pediatrics, Children’s Hospital of Philadelphia, Philadelphia, Pennsylvania, USA
2. Division of Cardiovascular Medicine, Department of Medicine, Hospital of the University of Pennsylvania, Philadelphia, Pennsylvania, USA

Supplemental Table 3: Factors affecting presence of tachyarrhythmia. On multivariable analysis, age at last ACHD contact and heterotaxy were independently associated with presence of tachyarrhythmia.

|  | Univariable |  |  | Multivariable |  |  |
| --- | --- | --- | --- | --- | --- | --- |
| Characteristic | Odds Ratio | 95% CI | p-value |  |  |  |
| Male | 1.08 | 0.70 – 1.68 | 0.718 |  |  |  |
| Age at last ACHD contact | 1.181 | 1.123 – 1.241 | <0.001 | 1.154 | 1.091 – 1.221 | <0.001 |
| Ventricular Morphology (ref = Single LV) |  |  |  |  |  |  |
| Single RV | 1.09 | 0.69 – 1.71 | 0.718 |  |  |  |
| Mixed Ventricular Morphology | 1.17 | 0.50 – 2.73 | 0.720 |  |  |  |
| Heterotaxy | 2.57 | 1.35 – 4.89 | 0.004 | 2.58 | 1.16 – 5.72 | 0.020 |
| Lateral Tunnel Fontan | 2.41 | 1.40 – 4.14 | 0.002 | 1.90 | 0.99 – 3.67 | 0.055 |
| History of Revision | 2.17 | 0.85 – 5.54 | 0.106 |  |  |  |
| Age at Fontan, Months | 1.012 | 1.006 – 1.019 | <0.001 | 1.003 | 0.994 – 1.011 | 0.543 |
